# ppb level detection of NO_2_ using a WO_3_ thin film-based sensor: material optimization, device fabrication and packaging†

**DOI:** 10.1039/c7ra13659e

**Published:** 2018-02-09

**Authors:** Chandra Shekhar Prajapati, Navakanta Bhat

**Affiliations:** Centre for Nano Science and Engineering, Indian Institute of Science Bangalore-560012 Karnataka India chandrashekhar@iisc.ac.in

## Abstract

In this study, we have investigated the thickness-dependent nitrogen dioxide (NO_2_) sensing characteristics of a reactive-ion magnetron sputtered tungsten trioxide (WO_3_) film, followed by morphological and electrical characterizations. Subsequently, the sensing material was integrated with an MEMS platform to develop a sensor chip to integrate with electronics for portable applications. Sputtered films are studied for their sensing performance under different operating conditions to discover the optimum thickness of the film for integrating it with a CMOS platform. The optimized film thickness of ∼85 nm shows the 16 ppb lower limit of detection and 39 ppb detection precision at the optimum 150 °C operating temperature. The film exhibits an extremely high sensor response [(*R*_g_ − *R*_a_)/*R*_a_ × 100 = 26%] to a low (16 ppb) NO_2_ concentration, which is a comparatively high response reported to date among reactively sputtered films. Moreover, this optimum film has a longer recovery time than others. Thus, an intentional temperature overshoot is made part of the sensing protocol to desorb the NO_2_ species from the film surface, resulting in full recovery to the baseline without affecting the sensing material properties. Finally, the optimized film was successfully integrated on the sensor platform, which had a chip size of 1 mm^2^, with an inbuilt micro-heater. The minimum power consumption of the microheater is ∼6.6 mW (∼150 °C), which is practically acceptable. Later, the sensor device was packaged on a Kovar heater for the detailed electrical and sensing characterizations. This study suggests that optimization of the sensing material and optimum operating temperature help to develop a highly sensitive, selective, stable, and portable gas sensor for indoor or outdoor applications.

## Abstract 2

In this study, we have investigated the thickness-dependent nitrogen dioxide (NO_2_) sensing characteristics of a reactive-ion magnetron sputtered tungsten trioxide (WO_3_) film, followed by morphological and electrical characterizations.

## Graphical abstract

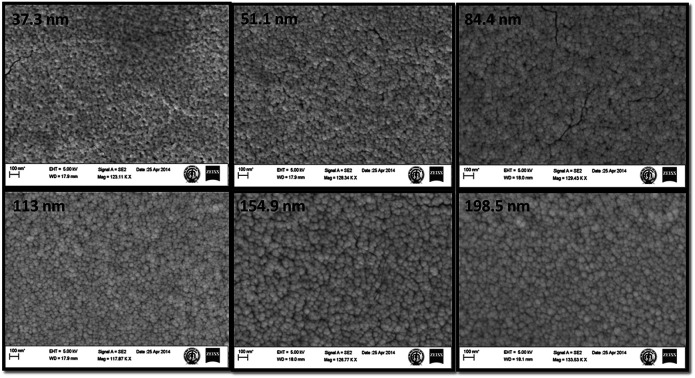

## 1. Introduction

Air pollution is one of the emerging problems in our surroundings. Thus, strict regulations on the emission of toxic gases require fast and highly selective gas sensors capable of detecting the sub-ppm level of gases. Carbon monoxide and nitrogen dioxide are the major pollutants, which play a major role in the formation of ozone and acid rain. Frequent exposure to NO_2_ levels higher than 53 ppb may cause an increase in respiratory illness.^1^ Therefore, inexpensive as well as simple fabrication procedures to develop sensors with high sensitivity, stability, and durability are in demand nowadays.

Thin films are more suitable for resistive-based gas sensors due to their high surface-to-volume ratio as the gas reaction is a surface phenomenon. Moreover, if the film morphology has a porous structure, gas molecules can easily react with the whole volume through the pores; this enhances the sensitivity. There are mainly two approaches for the improvement of the sensor sensitivity and selectivity. The first is the optimization of the sensing material growth/deposition conditions.^2–4^ The second is to quantify the operating conditions, such as operating temperature and bias voltage, of the sensor.^5,6^ In this study, the NO_2_ response is monitored by varying the thickness of a WO_3_ film with the impulse mode of temperature operation. Some reports have reported the effect of film thickness on sensor response.^7–14^ It can be understood that by controlling the microstructure shape and size of the WO_3_ film, the ppb level detection of NO_2_ can be achieved.^15–20^ In addition, not only an optimum sensing layer thickness helps to achieve a high response to test a gas but also the sensor operating conditions play an important role to define the overall sensor performance. In the past decade, WO_3_ nanostructures with large surface-to-volume ratios have been considered for gas sensing applications. Flower-like WO_3_ nanosheets, synthesized by calcining an acid-treated hydrothermal precursor, showed minimum 2 ppb NO_2_ level detection at a 90 °C operating temperature.^21^ Wojcik *et al.*^22^ studied the NO_2_ response of a drop cast-synthesized WO_3_ material and showed minimum 10 ppb NO_2_ detection at a 300 °C operating temperature. Triple-shelled WO_3_ spheres, prepared by ultrasonic spray pyrolysis, showed the minimum detection of 50 ppb NO_2_ at 100 °C, reported by Kim *et al.*^23^ A fully gravure-printed WO_3_-PEDOT:PSS nanocomposite-based NO_2_ sensor on a polyimide foil has been explored to detect minimum of 50 ppb NO_2_ at room temperature, reported by Lin *et al.*^24^ Recently, Zhang *et al.*^25^ reported 10 ppb NO_2_ detection at the 120 °C operating temperature using Fe-doped WO_3_ nanostructures synthesized by the hydrothermal method. Shen *et al.*^26^ have concluded that a Au-doped hierarchical WO_3_ microsphere nanostructure, prepared using the hydrothermal method, is capable of detecting a 1 ppm NO_2_ concentration at a 50 °C operating temperature. Although these nanostructures show a high response to NO_2_ in the sub-ppm concentration range, they are prepared through chemical route processes such as hydrothermal, drop cast, spray pyrolysis, which are not CMOS compatible. Although many studies have been reported on the physical deposition of a WO_3_ film for NO_2_ detection,^27–31^ no study has been reported on the realization of a sensor product from the optimization of a sensing film to the integration of the film with a MEMS platform. Thus, in this study, the sensing film of WO_3_ is optimized by varying the film thickness using a reactive-sputtering technique, followed by their sensing characterization to realize the best optimum film for highly selective response towards NO_2_ at the sub-ppb level. Later, using an MEMS platform with a low power integrated microheater, a large-scale production of a sensor chip, with a size of 1 mm^2^, is developed with integration of the optimized film. Packaging of the sensor chip on the header using a wire bonding process is conducted for easy integration with the electronics for real-time monitoring of sub-ppm levels of NO_2_ in air. The packaged sensor is highly sensitive and selective towards NO_2_ as further investigated.

To fabricate the highly sensitive and selective NO_2_ sensor device, the WO_3_ films of different thicknesses, deposited by an rf-magnetron-sputtering technique, were extensively investigated by sensing the characterizations at various operating temperatures ranging from 100 °C to 300 °C. Later, the optimized film was integrated with a CMOS compatible sensor platform, which was integrated with a micro-heater for the on-chip operation of the sensor device. In brief, sensor fabrication is mostly carried out with the help of photolithography and sputtering, followed by dry etch processes in reactive ion etching (RIE) and a deep reactive ion etching (DRIE) tool. The packaging is conducted on a Kovar header, followed by wire bonding for the easy handling of the sensor device, a prototype NO_2_ sensor.

## 2. Experimental

The conventional planar rf-magnetron sputtering system with a 3′′ target of tungsten in ambient oxygen is used to sputter WO_3_ films on top of the inter-digitated electrodes (IDEs). IDEs are patterned using photolithography, followed by Ti/Pt (10/80 nm) sputtering and a lift-off process, as shown in Fig. S1.† The distance between the target and substrate is maintained at 8.5 cm. An Ar gas flow of 300 sccm was maintained in the chamber by a mass flow controller, and the deposition pressure was kept at ∼6.3 mTorr. Before deposition, the chamber was evacuated to a pressure of the order of 10^−6^ Torr, and then, a pre-sputtering process was conducted to clean the target surface. The film thickness is controlled by adjusting the deposition time. The calculated average deposition rate of the WO_3_ film is ∼3.43 nm per minute, as shown in Fig. S2.† Film thickness was measured by a Dektak surface profiler and cross-sectional scanning electron microscopy (SEM). Surface roughness and grain size were analysed by atomic force microscopy (AFM). Surface morphologies were characterized by field emission scanning electron microscopy (FE-SEM). Finally, the as-deposited films were subjected to NO_2_ sensing characterization at different operating temperatures (100–300 °C) and gas concentrations.

## 3. Results and discussion

### 3.1. Structural and morphological characterizations of the WO_3_ films

X-ray photoelectron spectroscopy (XPS) is a widely used technique to investigate the chemical composition of thin films. The obtained XPS data of the WO_3_ films is shown in Fig. S3.† The study concludes that the sputtered films are pristine since there is no peak other than the characteristic peak for W and O. The doublet was observed at a binding energy of 33.9 eV and 37.0 eV corresponding to W 4f_7/2_ and W 4f_5/2_, respectively, from the core-level spectra of W_4f_, see Fig. S3.† This is in good agreement with other reported results.^32,33^ Therefore, it is clear that the W oxidation state is +6, which confirms the WO_3_ phase formation of the films. In Fig. S3(b),† the peak of O_1s_ core level is found at 530.87 eV, which is quite close to the value reported in the literature.^34^

**Fig. 1:**
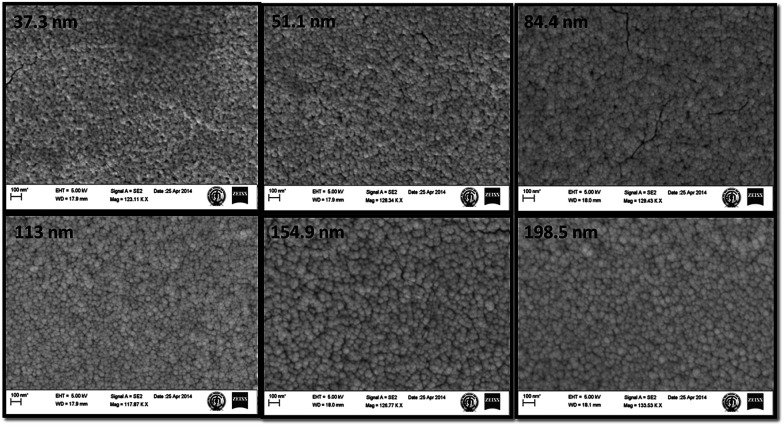
SEM images of the reactive sputter-deposited WO_3_ thin films.

**Fig. 2:**
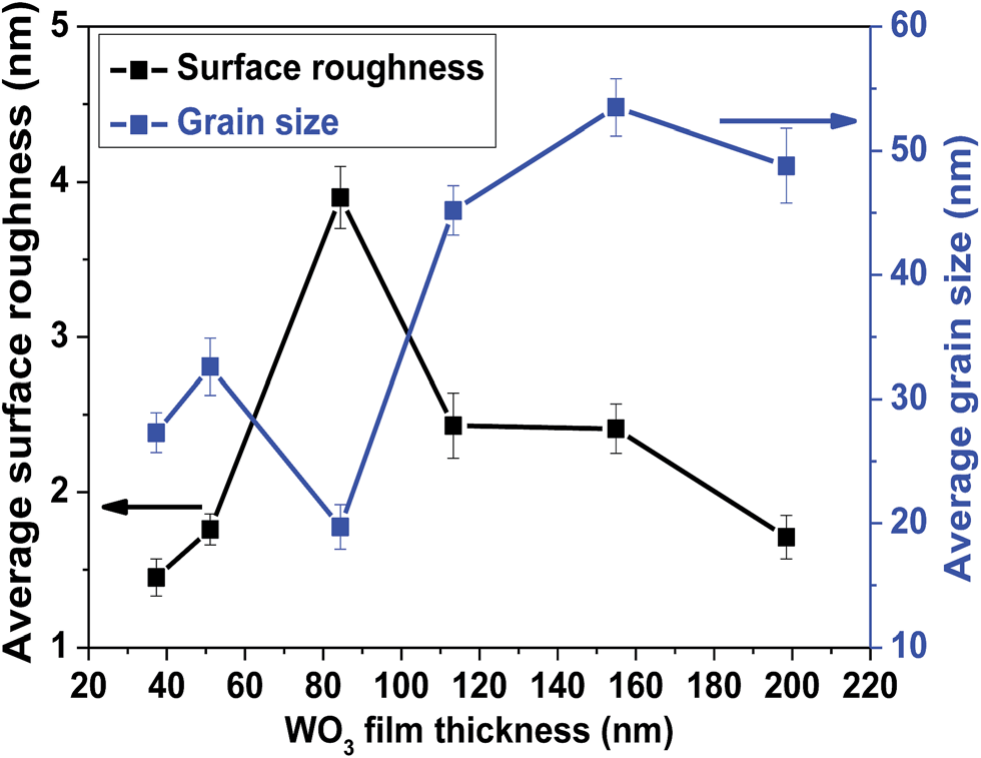
Variation of average surface roughness and grain size of the WO_3_ films with different thicknesses.

Surface morphologies of the as-deposited films of different thicknesses were studied using FE-SEM (Fig. 1). The topography of the films shows that the films have a porous structure with some black holes or zones on the surface. Films of lesser thickness have some minor cracks on the surface that provide direct conduits for gas molecules to flow inside the film; this may influence the sensor performance.^35^ The WO_3_ film of thickness ∼85 nm has a smaller grain size and higher surface roughness, as confirmed by the AFM analysis of the film grain size, as well as the surface roughness data, as shown in Fig. 2.

### 3.2. Electrical characterization of the WO_3_ films

The graph of the change in the electrical resistance of films with temperature in the range of 25–400 °C is shown in Fig. S4(a).† The resistance of the films, except the 37.3 nm, 113 nm, and 154.9 nm films, decreases rapidly with temperature up to 125 °C and thereafter begins to fall slowly up to 225 °C and afterward again decreases very slowly up to 400 °C. The overall trend of the film resistances indicates the semiconducting nature of the sputter-deposited WO_3_ films. In fact, two competing processes of thermal excitation of electrons and oxygen adsorption occur simultaneously. In the beginning, the decrease in the film resistance with temperature is because of the thermal excitation of electrons that dominates over the oxygen adsorption process. The slow decrease of film resistance in the temperature range from 125 °C to 225 °C is attributed to adsorption of atmospheric oxygen on the film surface. Herein, oxygen adsorption is not more favourable for the WO_3_ film; thus, the resistance of films decreases throughout the temperature range. A similar explanation has been reported by other authors.^36–38^ The inverse absolute temperature of the electrical resistance of the films is shown in Fig. S4(b).† Films exhibit two activation energies in different temperature ranges. The activation energy is calculated using the following relation:

1 *R* = *R*_o_ exp[(Δ*E*/*kT*)]

where Δ*E* is the activation energy, *R*_o_ is a constant, *k* is the Boltzmann constant, and *T* is the absolute temperature. The activation energies thus obtained are listed in Table S1,† which indicates two energies levels – one deep and one shallow near the bottom of the conduction band in the band-gap.

### 3.3. Nitrogen dioxide (NO_2_) sensing characteristics of the WO_3_ films

Room-temperature deposited films were tested several times at each operating temperature to guarantee the reliability of the sensing data. The sensor response (*S*) of the film is defined as the ratio of change in film resistance upon exposure to test gas to the film resistance in air (at same operating temperatures) and is given by the equation

2 *S* = (Δ*R*)/*R*_a_ × 100%

where Δ*R* is change in the resistance of the sensing film before and after exposure to the test gas and *R* is the initial resistance of film under an air atmosphere. To measure the sensing characteristics of thin films, the sensing setup used is shown in Fig. S5.† The film was mounted in a gas calibration chamber. The gas chamber had the ability to connect to the target gas cylinder along with the synthetic air (80% nitrogen and 20% oxygen) cylinder to set the appropriate concentration of the target gas using mass flow controllers (MFCs). The relative humidity was observed to be ∼45% inside the gas chamber during measurements. The resistance of the WO_3_ film was found to increase on exposure to NO_2_ gas due to the oxidizing nature of the gas. The sensing measurements were conducted under dry gas conditions.

**Fig. 3:**
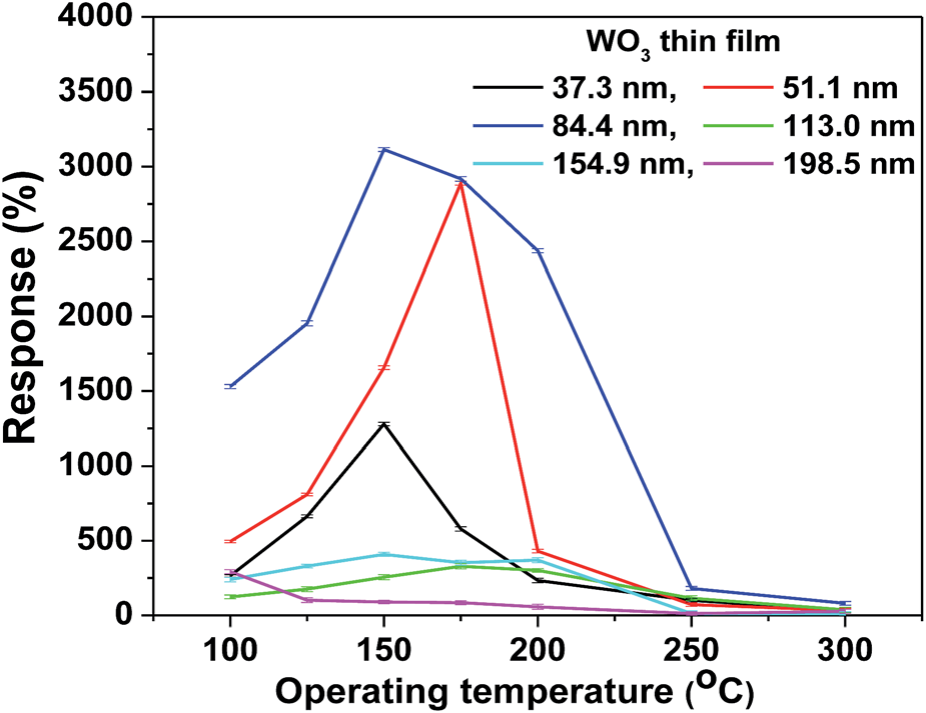
Response *versus* operating temperature plot of the as-deposited WO_3_ films for a 0.9 ppm NO_2_ gas concentration.

To determine the optimum thickness of the WO_3_ film for the maximum response to NO_2_, the gas sensing characteristics of different films towards 0.9 ppm NO_2_ were measured at different operating temperatures ranging from 100 °C to 300 °C, as shown in Fig. 3. It is well known that a high response depends not only on the optimum film thickness but also on the operating temperature. The present study concluded that the WO_3_ film thickness of ∼85 nm showed the highest response of ∼3102% to 0.9 ppm NO_2_ concentration at 150 °C, which was quite a low operating temperature.^13^

**Fig. 4:**
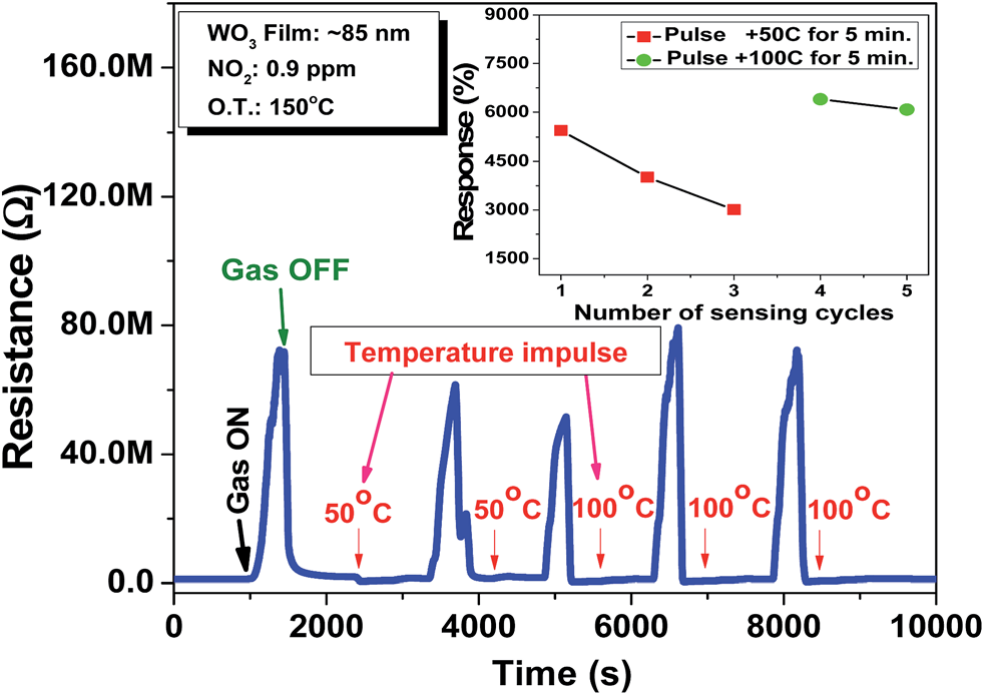
Impulse mode of operation of the sensor at an operating temperature of 150 °C for 0.9 ppm NO_2_ concentration (inset shows the comparison of sensor response with different temperature impulses).

To estimate the stability of the film response towards NO_2_, the ∼85 nm thick film is exposed multiple times to a 0.9 ppm NO_2_ concentration to quantify the resistance change of the film on each exposure. The film exhibits degradation in response after each exposure, as indicated by the drift observed in the baseline resistance of the film. The film is able to recover only ∼80% of the resistance, which is changed on exposure to NO_2_ gas. This may be due to the accumulation of incompletely oxidized gas molecules on the film surface. This results in an incomplete recovery of film resistance upon switching to synthetic air (NO_2_ exposure is off). To recover the sensor base line drift, the periodic shift to a higher temperature for a short duration is made the part of sensing protocol to desorb the gas molecules from the film surface.^2,39^ Thus, the temperature impulses of 50 °C and 100 °C of 50 seconds duration are implemented in between the sensing cycle, as shown in Fig. 4. From the initial two response cycles, it is clear that the recovery of film resistance is poor on impulse of 50 °C temperature, as can be seen from the obtained responses, as shown (red dots) in the inset of Fig. 4. This may be due to insufficient thermal energy for the gas molecules to desorb from the film surface. However after a temperature impulse of 100 °C, the base resistance is almost recovered, as shown (green dots) in the inset of Fig. 4. It can be concluded that the film shows a higher drift in sensor response treated with the impulse of 50 °C (red dots) as compared to the response drift in the case of a 100 °C temperature impulse (green dots). This kind of temperature treatment for a short duration is really effective to obtain the reproducible sensor response. In conclusion, temperature pulse of 100 °C is optimum to obtain the reproducible as well as the stable response towards NO_2_ using the WO_3_ thin film.

**Fig. 5:**
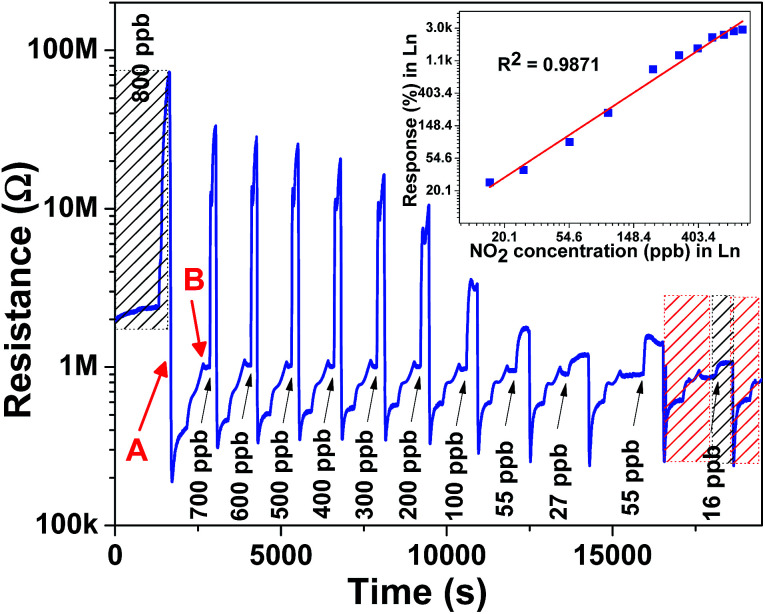
Different level NO_2_ sensing characteristics of the WO_3_ thin film at 150 °C operating temperature.

**Fig. 6:**
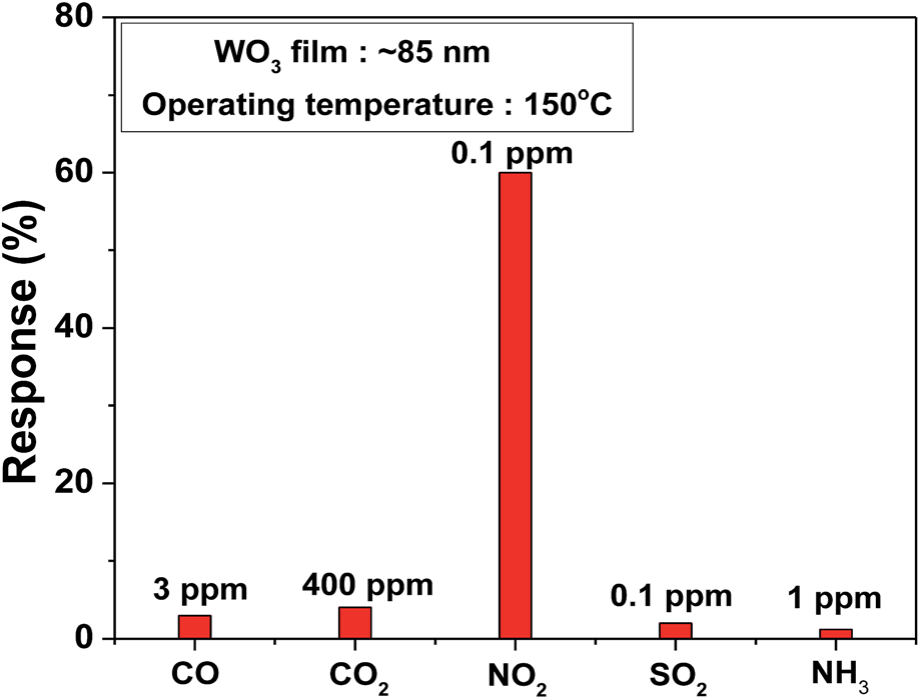
Selectivity check of the WO_3_ sensor at the operating temperature of 150 °C.

To estimate the low order of detection (LOD), NO_2_ gas concentration was tested from 16 ppb to 800 ppb at a 150 °C operating temperature with an optimum impulse of 100 °C temperature, as shown in Fig. 5. The film was able to detect 16 ppb [(*R*_g_ − *R*_a_)/*R*_a_ × 100 = 26%] NO_2_ concentration, which was comparatively low concentration than that reported in other studies.^40,41^ Moreover, the WO_3_ film shows a linear response to different NO_2_ concentration in the 16–800 ppb range and a detection resolution of 11 ppb for the optimum operating temperature (150 °C) with help of impulsive mode of temperature. Theoretically estimated LOD is 1.6 ppb obtained from the linear fit of response data of film shown in inset of Fig. 5. The comparison of present study along with responses observed by other researchers using different nanostructures of WO_3_ is shown in Table 1.^42–51^ The present study on the WO_3_ thin film-based NO_2_ sensor concludes that sub-ppb level NO_2_ detection with high sensitivity and selectivity can be obtained by simple reactive-ion sputtered technique, a scalable process. The WO_3_ film selectivity towards NO_2_ was tested in the presence of CO, CO_2_, SO_2_, and NH_3_ gases at 150 °C. The study clearly indicates the high selectivity of the film towards NO_2_ among other gas species, as represented in Fig. 6.

**Table 1:** Comparison of NO_2_ sensing characterisations with different nanostructures of WO_3_ materials

Sensing material/morphology	Synthesis method	NO_2_ (ppm)	Sensor response	Operating temperature (°C)	Response time (s)	Selectivity	Ref.
Flower-like WO_3_ nanosheets	Acid treated hydrothermal	2–80 ppb	*R*_g_/*R*_a_ = 12.8 (2 ppb)	90	—	Cl_2_, CO, H_2_S, NH_3_, C_2_H_5_OH, CH_3_COCH_3_	21
WO_3_ powder	Drop cast method	0.01–0.25	*R*_NO_2__/*R*_air_ × 100% = 1.2 (10 ppb)	300	40 for 100 ppb	CO, CH_3_COCH_3_, H_2_S, H_2_, CH_4_	22
Multi-shelled WO_3_ yolk–shell spheres	Ultrasonic spray pyrolysis	50 ppb	*R*_g_/*R*_a_ = 100 (50 ppb)	100	245 for 50 ppb	CH_3_COCH_3_, C_2_H_5_OH, CO, NH_3_, C_7_H_8_	23
WO_3_–PEDOT:PSS nanocomposites	Gravure-printed technique	50–200 ppb	*R*_g_/*R*_a_ = ∼1.2 (50 ppb)	RT	45.1 for 50 ppb	NH_3_, H_2_, CH_3_COCH_3_, C_2_H_5_OH, CH_3_OH	24
Fe-doped WO_3_ mesoporous	Facile method	10–1000 ppb	*R*_g_/*R*_a_ = 1.3 (10 ppb)	120	52 for 10 ppb	H_2_, CO_2_, CH_3_COCH_3_, CO, NO_2_	25
Au-doped WO_3_ microspheres	Hydrothermal	1–10	*R*_g_/*R*_a_ = 1 (1 ppm)	50	75 for 5 ppm	—	26
WO_3_ thin film	Spray pyrolysis	1–750	(*R*_a_ − *R*_g_)/*R*_a_ = 0.1 (1 ppm)	125	0.6 for 250 ppm	—	42
3D WO_3_ nanocolumn bundles	Hydrothermal	1–320	*R*_g_/*R*_a_ = 2.06 (1 ppm)	110	23 for 10 ppm	—	43
WO_3_ hierarchical	Hydrothermal	1–20	*R*_g_/*R*_a_ = 7 (1 ppm)	100	41 for 5 ppm	—	44
Au-decorated WO_3_ nanodomes	Soft templates	0.3–5	(*R*_g_ − *R*_a_)/*R*_a_ = 361 (5 ppm)	250	63 for 5 ppm	CH_3_COCH_3_, C_2_H_5_OH, NH_3_, CO, H_2_, C_6_H_6_	45
3-D WO_3_–rGO hybrid nanostructure	Hydrothermal	5–200	*R*_g_/*R*_a_ = 4.3 (10 ppm)	90	4.1 for 10 ppm	—	46
3DOM WO_3_/Li	Colloidal crystal template	0.5–1	*R*_g_/*R*_a_ = 55 (0.5 ppm)	25	300 for 0.5 ppm	C_2_H_5_OH, NH_3_, CH_3_COCH_3_, C_6_H_4_ (C_2_H_5_)_2_	47
Villi-like WO_3_ nanostructures	RF sputter	0.2–5	*R*_NO_2__/*R*_Ethanol_ = 500 (5 ppm NO_2_/50 ppm CH_3_COCH_3_)	250	—	C_2_H_5_OH, CH_3_COCH_3_, NH_3_, C_7_H_8_, CO	48
WO_3_ thin film	Red-ox route HFCVD	5–750	*R*_g_ − *R*_a_/*R*_a_ = 32 (5 ppm)	250	—	—	49
WO_3_ nanorods (annealed at 500 °C)	Glancing angle dc magnetron sputtering	0.1–2	*R*_g_/*R*_a_ = ∼27 (2 ppm)	250	—	—	50
WO_3_ nanoparticles	Sol–gel method	5–150	(*R*_g_ − *R*_a_)/*R*_a_ × 100% = 16 (5 ppm)	200	24 for 100 ppm	NH_3_, H_2_S, C_2_H_5_OH, Cl_2_, CH_3_OH	51
WO_3_ thin film	Rf reactive-sputter	16–3 ppm	(*R*_g_ − *R*_a_)/*R*_a_ × 100% = 26 (16 ppb)	150	200 for 16 ppb	CO, CO_2_, SO_2_, NH_3_	Present work

The present investigation of WO_3_ thickness-dependent NO_2_ characteristics suggest that a film thickness of ∼85 nm is optimum to achieve a highly sensitive and selective NO_2_ sensor, which significantly shows the sub-ppb range detection with a quick response and recovery time. Furthermore, to realize the prototype NO_2_ sensor, we fabricated a sensor device inbuilt on-chip-integrated microheater to control the operating temperature of the WO_3_ film using MEMS surface micromachining processes, as explained hereinafter. The detailed optimization of the fabrication process of the sensor device is described elsewhere.^52^

### 3.4. Fabrication of the NO_2_ sensor device

**Fig. 7:**
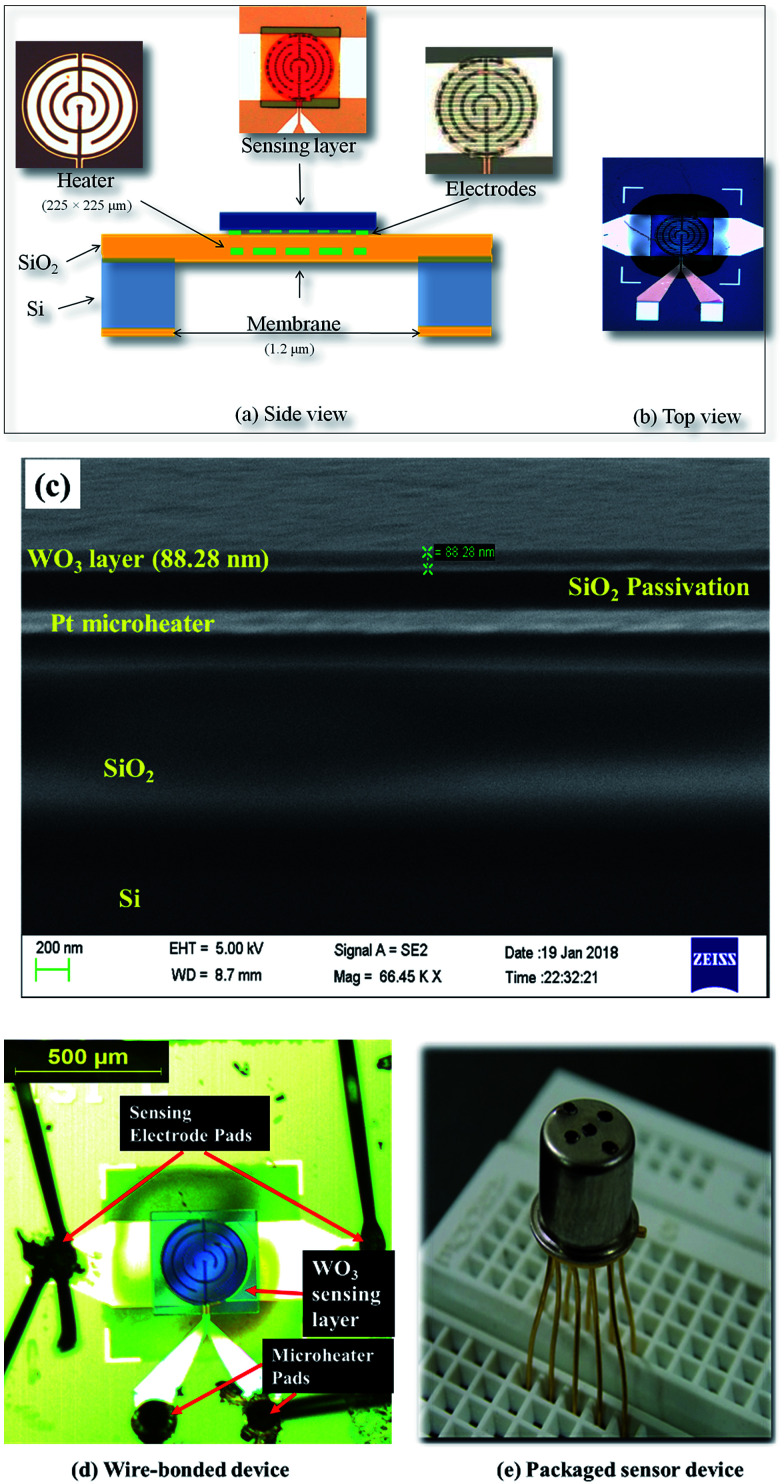
(a) Side-view of the micro-sensor, (b) top-view of the micro-sensor, (c) cross-sectional SEM image of the sensor chip, (d) device after wire-bonding, and (e) image of the packaged sensor.

A 4′′ wafer was cleaned prior to SiO_2_ deposition first by piranha solution and then dipped in hydrofluoric acid followed by washing with DI water and drying in nitrogen. The front side SiO_2_ (1 μm) is used to build the sensor device, and the back side SiO_2_ (1 μm) is used as a mask for backside Si etching in deep reactive ion etching (DRIE). On top of SiO_2_, Ti/Pt (10/80 nm) is sputtered to a pattern microheater structure. Then, 200 nm PECVD SiO_2_ is deposited on top of the microheater (200 μm × 200 μm) to serve as an insulator between the micro-heater and sensing electrodes. Sensing electrodes of sputtered Ti/Pt (10/50 nm) are fabricated on top of the microheater. Later, as optimized, ∼85 nm thick WO_3_ film is deposited onto the sensing electrodes. Finally, bulk Si from the back side of the microheater is dry etched to form an air cavity to reduce the power consumption of the microheater. The deposition and lift-off process of the WO_3_ sensing material is conducted using sputtering and photolithography. The schematic of the fabricated sensor device is shown in Fig. 7(a and b). To estimate the WO_3_ sensing layer thickness, a cross-sectional SEM image of the fabricated sensor chip is shown in Fig. 7(c), which indicates the sensing layer thickness of ∼88 nm on top of the sensor chip stacks. To check the response of the fabricated sensor device towards NO_2_ gas, the measurement is obtained from the packaged sensor, as shown in Fig. 7(d and e).

**Fig. 8:**
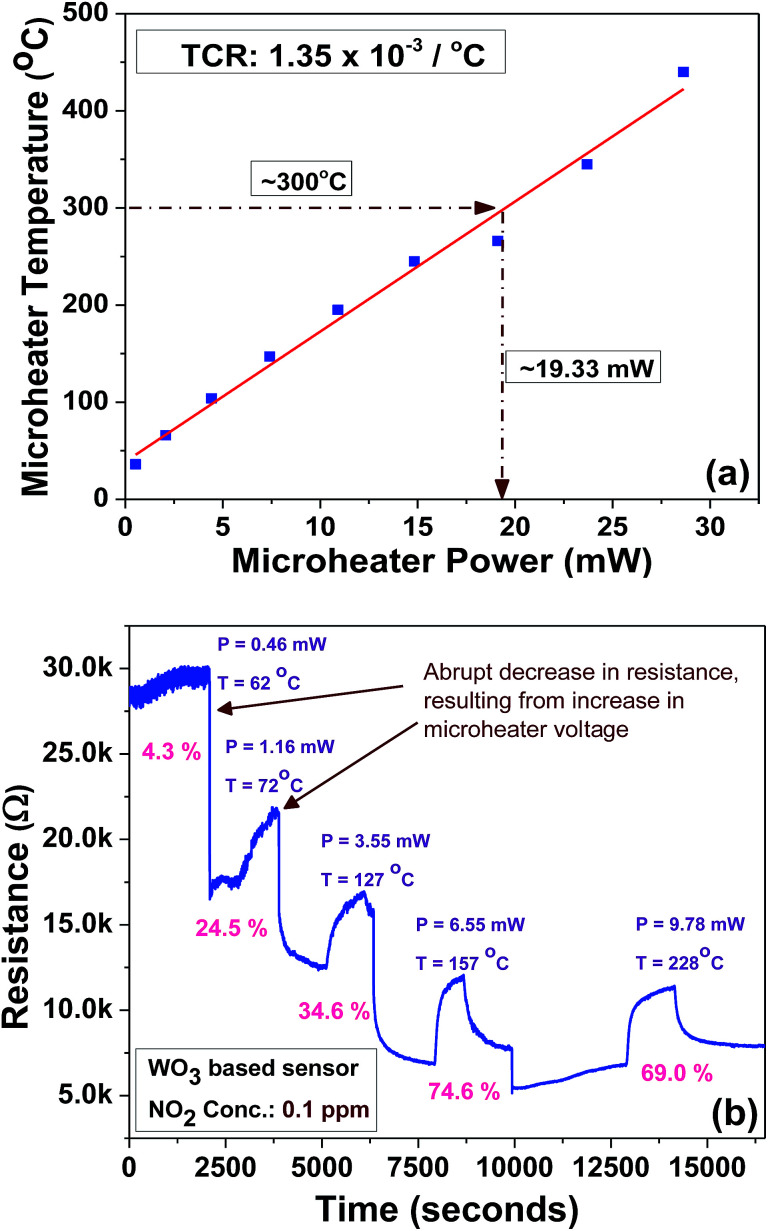
(a) Microheater power consumption *versus* temperature plot. (b) Sensor response at different operating temperatures at a fixed NO_2_ concentration (0.1 ppm).

**Fig. 9:**
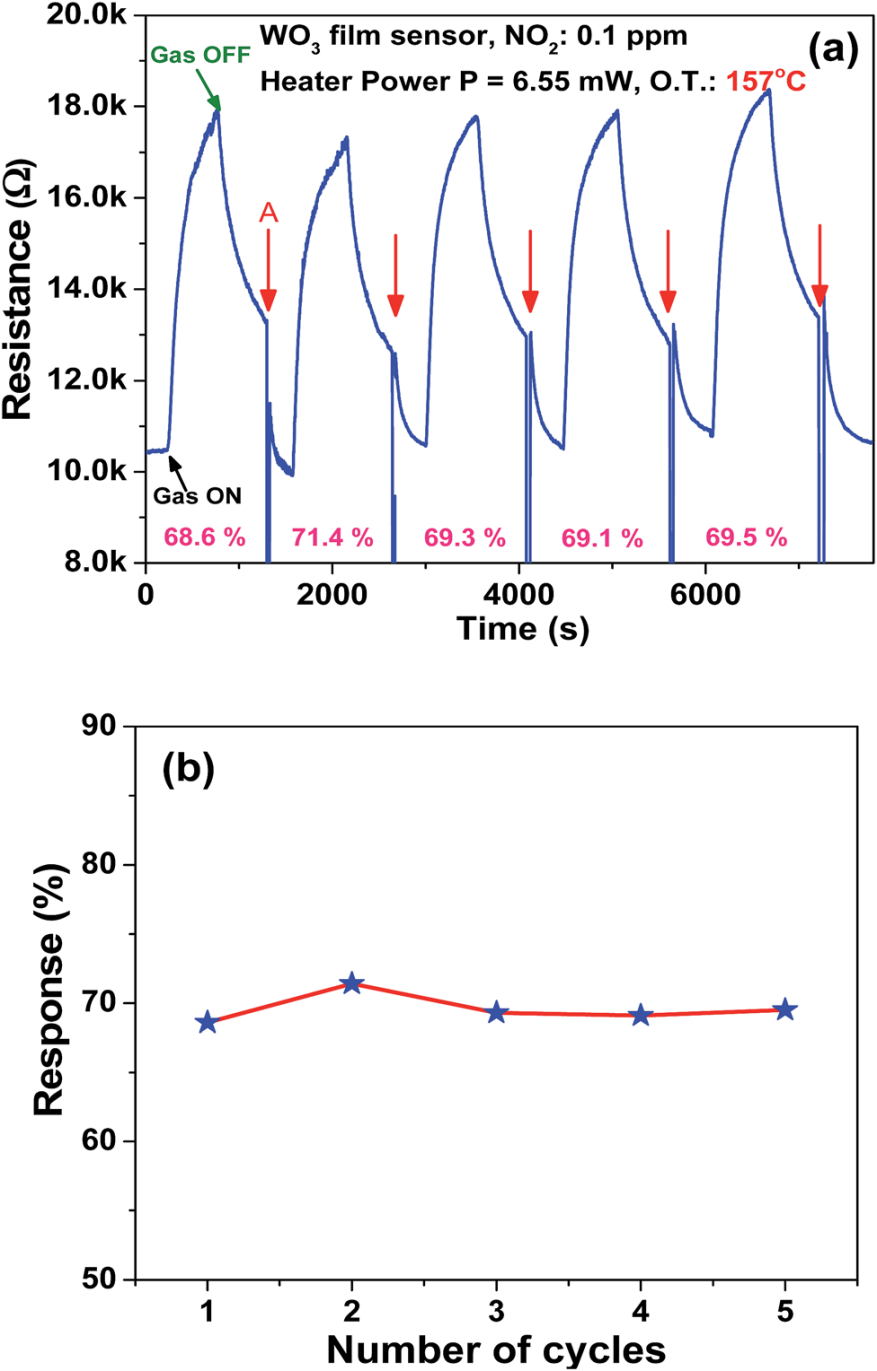
(a) Sensor's repeatable response characteristics at a fixed operating temperature of 157 °C and gas concentration of 0.1 ppm (symbol A indicates an abrupt decrease in resistance, resulting from a rapid increase in heater voltage) and (b) sensitivity *versus* number of cycles curve.

**Fig. 10:**
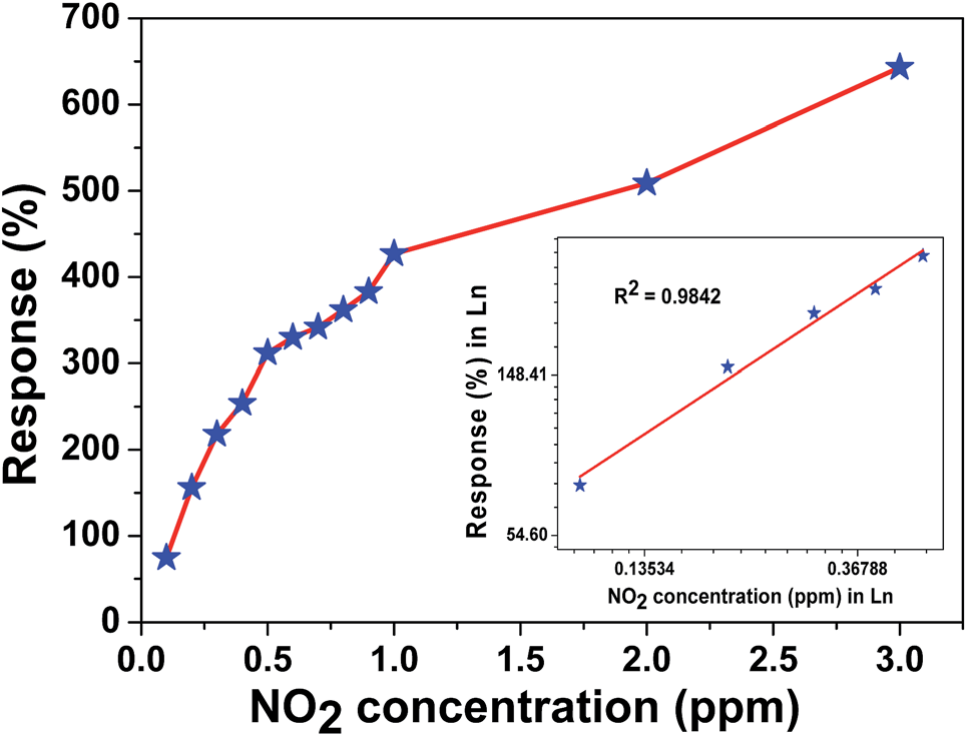
Response *versus* NO_2_ concentration plot of the fabricated sensor.

The microheater characterization was conducted to calculate the heater power consumption to achieve different temperatures from the microheater, as shown in Fig. 8(a). The calculated temperature coefficient of resistance (TCR) is 1.35 × 10^−3^ °C^−1^. Initially, the gas sensitivity is measured under the fixed (0.1 ppm) NO_2_ concentration at different operating temperatures, ranging from 62 °C to 228 °C, to know the optimum operating temperature to achieve a high response, as shown in Fig. 8(b). The sensor device shows the high response of ∼74.6% at 157 °C, which requires a power of ∼6.55 mW. To examine the repeatability of the fabricated sensor, the sensor is exposed multiple times to a fixed NO_2_ concentration (0.1 ppm), as shown in Fig. 9(a). The sensor shows an almost repeatable response, but the recovery of the sensor's base resistance is still an issue. Thus, to overcome this issue, the sensor was operated with an impulse mode of temperature by increasing the heater voltage for 20 seconds, which increased the operating temperature by ∼100 °C. As a result, the sensor response is almost repeatable Fig. 9(b). Fabricated sensors are also exposed to different NO_2_ gas concentrations from 0.1 ppm to 3 ppm under the same operating conditions. For a very low concentration range from 0.1 ppm to 0.5 ppm, the sensor shows a rapid change in response. However, for high concentrations, the increase in response is comparatively slow, as shown in Fig. 10. The rapid change at low concentrations may be because gas-molecules obtain enough thermal energy to react with the sensor surface; this leads to a fast reaction at the sensor sites. On the other hand, with an increase in gas concentration, the gas molecules may be covering the sensor surface very fast; this leads to a slow increase in response. Moreover, the fabricated NO_2_ sensor shows a nearly linear response in the concentration range from 0.1 ppm to 0.5 ppm with a detection resolution of 100 ppb for the optimum operating conditions, as shown in the inset of Fig. 10. The results indicate that the sensor is capable of detecting a NO_2_ gas concentration as low as 100 ppb. In fact, many models have been proposed to describe the sensitivity of the semiconducting metal oxide; thus, it can be represented empirically.^53^ The lowest order of detection of the NO_2_ sensor is 0.8 ppb, which is calculated by a linear fit of the sensor response data in the concentration range from 0.1 ppm to 0.5 ppm, as shown in the inset of Fig. 10.

We have monitored the fabricated NO_2_ sensor response characteristics to evaluate the sensor reproducibility and stability for a period of more than 6 months to estimate the sensor life. The as-fabricated sensor is found to be very stable during this period. Thus, we propose that the present sensor is a promising candidate for real-time monitoring of NO_2_ gas in air.

## 4. Conclusions

In conclusion, film surface morphology plays an important role in deciding the sensing characteristics of thin film-based sensors. XPS analysis of sputter-deposited films shows the desired chemical states. SEM images show that films have a porous microstructure with small cracks, which helps to enhance the sensing reaction because of the deep interaction of gas molecules with the film. The impulse mode of temperature is implemented successfully to produce a highly stable and reproducible sensor response. With these sensors, a detection limit of 16 ppb for NO_2_ is achieved. This is the lowest detectable concentration with this pristine metal-oxide semiconductor to date. Sensors show high selectivity as well as sensitivity to NO_2_ gas.

The NO_2_ sensor device is fabricated successfully using an MEMS platform and tested under different operating conditions to evaluate the performance of the sensor. An impulse mode of temperature is found to be effective to recover the baseline drift in NO_2_ sensor resistance. The choice of sensor elements on a single diaphragm exhibits fairly good cross-sensitivity, long-term stability, as well as reproducibility towards NO_2_ gas detection.

## Conflicts of interest

There are no conflicts to declare.

## Supplementary Material

RA-008-C7RA13659E-s001
