# Supplementary material for: ppb level detection of NO2 using a WO3 thin film-based sensor: material optimization, device fabrication and packaging
Source: RSC Adv. 2018 Feb 9;8(12):6590–9. doi: 10.1039/c7ra13659e (PMC9078366; doi:10.1039/c7ra13659e)
Supplement: RA-008-C7RA13659E-s001 [file RA-008-C7RA13659E-s001.pdf]

Electronic Supplementary Material (ESI) for RSC Advances.
This journal is © The Royal Society of Chemistry 2018

Foot Notes

S.M. 1 Interdigitated electrodes (IDEs) patterned using photolithography, followed by sputtering process.

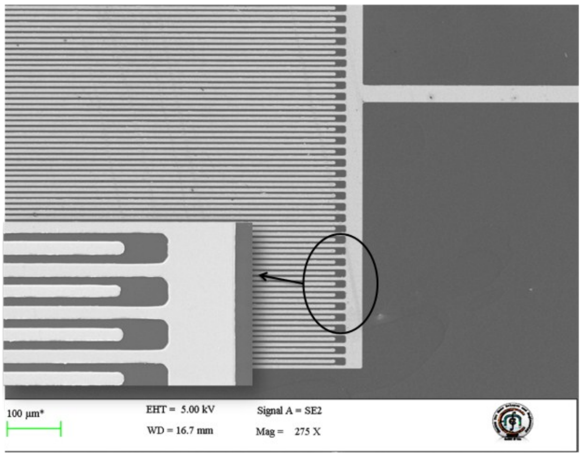

The NO_2 sensing characterization of WO_3 films, deposited on IDEs structure, are performed using rf-magnetron sputtering technique

S.M. 2 Average deposition rate of reactive sputtered WO_3 film at optimum conditions.

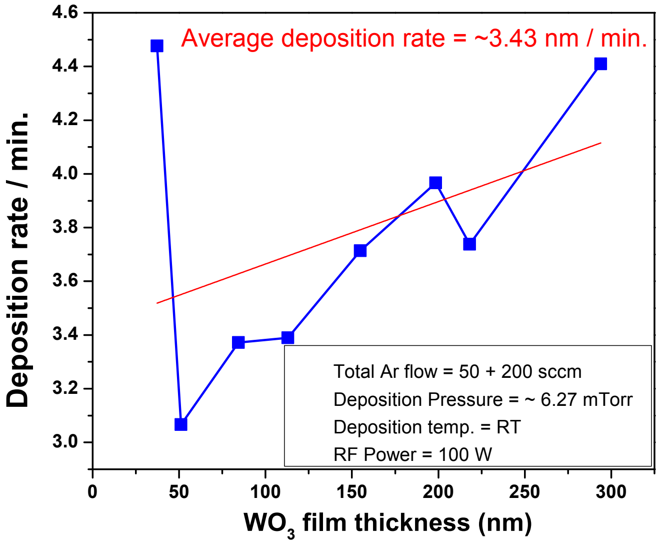

Films are deposited using reactive-ion sputtering of W target on SiO_2-/Si substrate to investigate the effect of thickness on NO_2 sensing characteristics. Film thickness is calculated by using dektak profiler and cross-sectional SEM images. The average deposition rate at optimum conditions is ~3.43 nm per minute.

S.M. 3 XPS spectra of WO_3 thin films; (a) W_{4f} doublet core level and (b) O_{1s} core level.

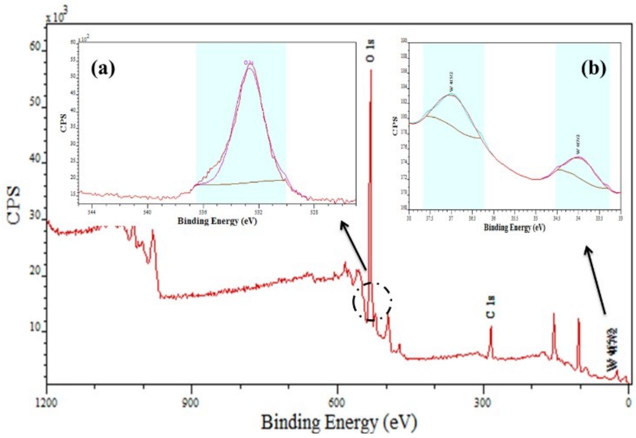

The XPS spectra of WO_3 film shows the pure formation of rf-magnetron sputtered WO_3 film.

S.M 4 Temperature-dependent resistance; (a) Variation of the electrical resistance of WO_3 films with temperature and (b) inverse absolute temperature of the electrical resistance of the WO_3 films.

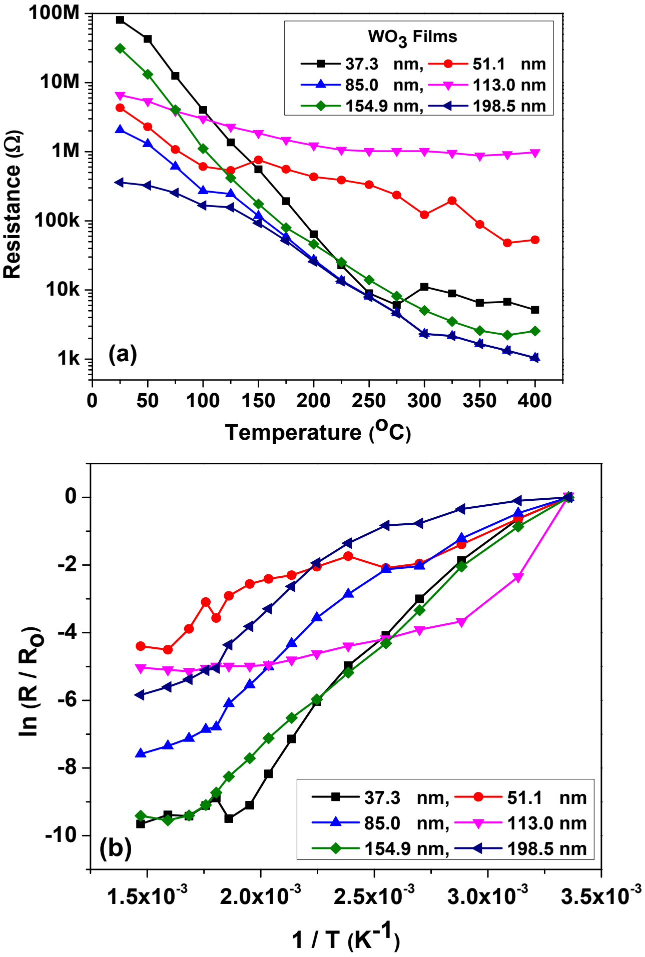

WO_3 films of different thicknesses are studied in the temperature range from room temperature to 400°C to calculate the activation energies. Films are found to be in semiconducting. Films exhibit two activation energies, which indicates two energies levels – one deep

and one shallow near the bottom of the conduction band in the band-gap.

S.M 5 Experimental setup used to study the sensing response of films and sensor devices.

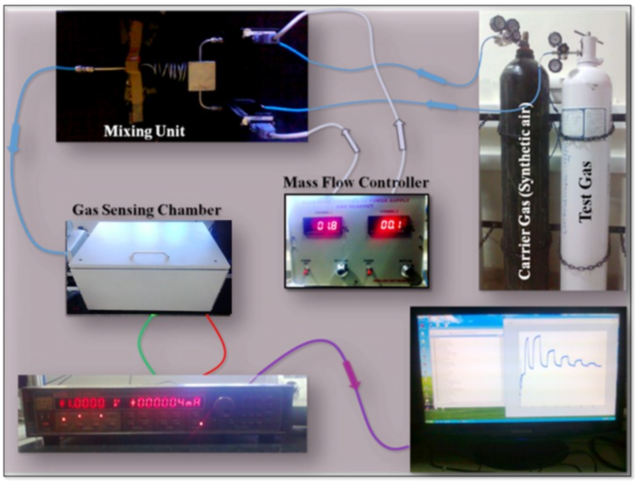

Two MFCs were used to set the gas flows for synthetic air and the target gas and a static gas mixer for uniformly mixing of target gas with synthetic air before entering the gas chamber. The total gas flow rate was fixed to 500 sccm for each concentration. The exposed concentration range is below the permissible exposure limit (PEL) of NO_2 gas. The real-time monitoring of sensor's signal is recorded by using the Matlab R2010a program, where the data acquisition is done every second. A Eurotherm feedback system, with a thermocouple, controls the on/off time of an incandescent lightbulb located inside a graphite block, and thereby controlling the operating temperature of the sensor.

Table

Table S1: Activation energies of WO_3 thin films

WO_3 Films	Activation energy ΔE (eV)
~37.3 nm	0.438 eV (Temperature range: 25 – 125°C), 0.760 eV (Temperature range: 150 – 225°C), 0.173 eV (Temperature range: 300 – 400°C)
~51.1 nm	0.234 eV (Temperature range: 25 – 100°C), 0.139 eV Temperature range: 150 – 225°C), 0.372 eV (Temperature range: 300 – 400°C)
~85.0 nm	0.238 eV (Temperature range: 25 – 100°C), 0.517 eV Temperature range: 125 – 225°C), 0.208 eV (Temperature range: 300 – 400°C)
~113.0 nm	0.165 eV (Temperature range: 25 – 100°C), 0.142 eV Temperature range: 150 – 225°C), 0.280 eV (Temperature range: 300 – 350°C)
~154.9 nm	0.448 eV (Temperature range: 25 – 100°C), 0.452 eV Temperature range: 125 – 175°C), 0.308 eV (Temperature range: 300 – 375°C)
~198.5 nm	0.094 eV (Temperature range: 25 – 100°C), 0.302 eV Temperature range: 125 – 225°C), 0.206 eV (Temperature range: 300 – 400°C)
